# Supplementary figures and images for: Identification of aluminum-activated malate transporters (ALMT) family genes in hydrangea and functional characterization of HmALMT5/9/11 under aluminum stress
Source: PeerJ. 2022 Jun 24;10:e13620. doi: 10.7717/peerj.13620 (PMC9235816; doi:10.7717/peerj.13620)

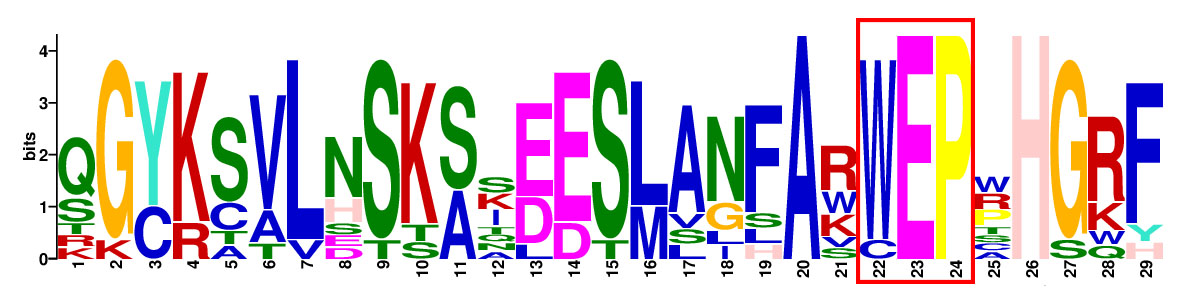

Supplement: Supplemental Information 1 [file peerj-10-13620-s001.jpg]
