# Supplementary material for: Identification of aluminum-activated malate transporters (ALMT) family genes in hydrangea and functional characterization of HmALMT5/9/11 under aluminum stress
Source: PeerJ. 2022 Jun 24;10:e13620. doi: 10.7717/peerj.13620 (PMC9235816; doi:10.7717/peerj.13620)
Supplement: Supplemental Information 2 [file peerj-10-13620-s002.docx]

>HmALMT1

MESSNQERVGPHKCGWAWFKGLIEKLRSYVVDFGKYSKKLGEEDPRRIIHSLKVGLAISI

VSLIYYFDALFEGFGISAMWAVMTVVVVFEFSVGATLGRGLNRGIATLLTGTLGVGAHRL

ARFSGEACEPIILGLFVFIIAVMVTFLRFFPRMKARYDYGLLIFNLTFSLVAVSGYQDDE

VLDIAHKRLSTILIGGAVAVIICICICPVWTGTDLHNQVATNLEKLAIFLEGFGGEYFTN

SEDKESENDKSSMQGYKSVLNTKSIEESMVNLAKWEPRHGWFRYRHPWNQYLEIGALTRQ

CAYTIETLNNYLNSEIKTPIEIREKIKEPCMKMSSESSLALKELAMEIKEMTQSSNAKTH

IAHSKIAAKNLKSKFQTGLWRDSDQLLGLIPATTVASLLLDVVICTDKIGKSVHELASLA

RFKTSDAAIKSKELKLNQKELLKSCSGITIDGSSNYSLPNSGGE

KSCSGIQGHHH

>HmALMT2

MVIESPTQKKTSLIIAFMCSWAKALLEKFKAKTIEITKKTKKIGKDDPRRIIHSLKVALA

LTLVSMFYYLRPLYDGFGAAGMWAVLTVVVIFEFSVGATLCKGLNRGLATFLAGAFGIGA

NYLANLLGETGEPILLGLLVFILGAASTFTRFFPVIKARYDYGAVIFLLTFSLVAVSGYR

VDEIIELAHQRLSTIIAGGAICIVVSIFICPVWAGEDLHNLLVLNLEKLASFLEGFGGDY

FKFPEDGGSTMISKDDKSYLQGYKSVLNSKSSEESLANFAWWEPCHGQFRFRHPWKQYLK

IGGLTRQCAYKVEALSGYLHFDIQEPSEFQRKIQESCYKMSSELAKALKELASVIKTMSC

PSCSNIHIEKSKIAVDELKTALLTVSSSLEKKRAY

>HmALMT3

MKGKKGSFEINIPNVTKAKMPETGKGLDENGFSFKSWIFSVWEFAKEDSNRVTFSLKVGL

SVLLVSLLILLRAPYQIFGNNIIWSIITVAIMFEYTVGATFNRGLNRALGSLLAGILAIA

VAQLALRTGHVAEPVIIGISIFLIGAITSFMKLWPSLTPYEYGFRVVLFTYCLIIVSGYR

MGNPLKTAMERLYSIAIGGIVAVLVNVLVLPIWAGEQLHKELVDSFDSVAASLEECVRKY

LEDDGSEHPEFSKTVMDEFPDEPAYRKCRSTLSSSAKLESLAISAKWEPPHGRFKHFFYP

WSEYVKVGAVLRYCAYEVMALHGVLHSEIQFKLGGTQMFDSIAPYNLRITFQKEIQEATT

QAAELVRCLGKDISNMKRSLKTTLLKRVHSSTERLQNAINMHSYLLTTNHEPPESDSSKP

LPTLSHTHSSTLYDLSNKLAELDNTNPDQSSNQPTQRAPSESLQTESYHETMRKQSRRLH

SWPSREVDDYEEEGGFSTDFIPRMQALESTAALSLATFTSLLIEFVARLDHLVEAVDVLC

KMAKFKHEVL

>HmALMT4

MRKVIHSIKVGIALVLVSLFYLLDPLYRQVGENAMWAIMTVVVVFEFFAGATLSKGLNRG

IGTILGGGLGCFAAILADKVGGKIGNPIVVGTSVFIFGAVATYARLIPSIKRRYDYGVLV

FILTFSLVVVSGLRADKVMEIARERLSTIGMGFAVCIFTSLLIFPTWASDELHYSVASKF

ENLATCIEGSLEEYFTVVNEKENHPIKANFTGCKSVLHSKSIDESLANFARWEPWHGKFG

FSYPWEKYLQIGEVLREIAATTLSLKGCLQSPRQPSPMLRQLIKEPCEAVGSSLAWNLRE

LGESIAKMKRYQIANKALMAQKLQPLKLELCLVTSPSKLGTDDQESTHDEGLAMSSFVFS

LMEMVDKLEILAEKVEELGDLANFDTN

>HmALMT5

MSSTVVICIPKEDGILIAPKQQKKIQFSVLITVIISSIRDKKYCNKDVRKVIHGVKVGIA

LVLVSLLYLLDPLYKQFGNNAMWAIMTVVVVFEFFAGATLSKGLNRGIGTIVGGGLGCLV

AILADKVGGIGNPITVGSFVFIFGAAATYSRLVPSIKRRYDYGVMIFILTFNLVVVSGVR

ADKIMEIASDRLSTIGMGFAVCIFTSLLIFPMWASDELHYSFAYKFENLASCIEGCLDEY

FSVVDNEKEKKAPANYFSGCKSVLHSKASDESLANFARCEPWHGKFGISYPWQKYLEIGE

VLRELAATILSLKGCLNSSRQPSSTLRHSIKEPCEAIGSSLAWNLRELGESIMKMRRCRP

YKDSMAEKMESMKLDLGLIKTPSKLGTEENGEAGLAIASLVFLLMEIIEKMETLAEKVEE

LGELASFQTDHQQLTVCT

>HmALMT6

MYSGYRTAVESTSQEDSLLGFAVWEPPHGRYKMFGYPWSDYVKVSGALRHCAFMVMAMHG

CILSEIQAASELRQIFLNEIQRVGTEGAKVLHELGNKIEKMEKLNPEDLLCKVHEAAEEL

QMKIDKKSYILVNSANWESGRRPKEFQDSDSFHEVKDSENKQIVINSISELGLNMRSAAN

LRNWDSSHNPNMSINPSTQWGSSEDMFKKPIAWPSRFSLLGDTILNEREVRTHESASALS

LATFTSLLIEFVARLQNLVDSFEELSEKANFKEPVDPNASKDSLEMELSSDTVFTACIAK

LGGSEKDKNSEENHRWESSGIAICLDYDRPYGLMTERQVSNGPNLTHTPRILPLVLQDRN

EKVELRSNSRRQTREIATNLEIVWHFPLICLAVFGKSNETAGT

>HmALMT7

MRPLYEGVGGNAMWAIMTVVVVFEYTVGATLCKCVNRALGTFLAGCLGIGVHWVATQPEQ

KFEPIILGVSVFLLASAATFSRFIPSVKARFDYGAVIFILTFSLVSVSGYRVDELFELAQ

CRVSTIAIGTSICLLISMLFCPIWAGNELHYLTTRNLDKLADSLDGCVALYFKWDKKGNS

NEEDCSKKLQGYKCVLNSKAAEESMANFARWEPTHGRFNFGHPWKQYLKIGSAIRSCAYC

IETLNGCFNSELQAPDFLKEHFGDVCKKLSSNSSNVLKELAVTIKTMTKSSKMDLSVGEM

NFAVEELQNALKTLPNQLIPPPLSPSKASNDQKEEPIMKGTIVPIMDVLPMATLVSLLVE

IAVRIDGIVNEVDDLSGQTQFMPASDEKSKLTQIITDDEDHEENRQKV

>HmALMT8

MEIESADHKKIGLMTTCGCHWIKALFRKFKFKTQEISRKTKNIAQADPRRIIHSLKMALA

LTLVSMFYYLRPLYDSFGSAGMWAVLTVVVIFEYTVGATLSKGLNRGFATLLGGSLGIGA

EYLAGLLGEKGEPILLGFSVFLLASASTFSRFFPHIKARYDYGVLIFILTFSLVAVSGYR

VDKIVELAHQRLSTIIVGGATCMIISIFLCPVWAGEDLHNLVALNIEKLASFLEGFGGEY

FKISEDGDNSVVSKDDKSFLQGYKSALNSKSNEDSLSNFAWWEPSHGRFGFRHPWKQYLK

IGGLSRQCAYKLEALSGYINSDIHVPSEFQRKIQDPCTKISSESGKALKELASAIKMMTC

PSSAQTHIKNFQKAIIELNTALEASLLDESNLMETIPAITVASILIEITNWVEKIAESVN

ELSQLAHFKNLELGVQPAKPQVLSRGAIKPVSDNDGDGDHVVIVVHGTNNDTPPPEKKDP

QAPNMGV

>HmALMT9

MAAKLGSFRYSFKEKRERERFLLSYKGGYSELAGGFAIDEVEEKNSRFCSCGSIRDRFAL

SWKFLRGVAVKALEMGQSDPRKIIFSAKMGLALMLISLLIFLKEPSKDLSRYSVWAILTV

VVVFEFSIGATLSKGFNRGLGTLIAGGLALGMAELSQLAGESEEVVIIISIFITGFLATY

AKLYPTMKAYEYGFRVFLLTYCFIMVSGYRTREFIHTAVTRFLLIALGAGVCLVVNICIY

PIWAGEDLHNLVAKHFAGIATSLEGCVNEYLNCVEYERIPSKIITYQASDDPMYSGYRTA

VESTSQEDSLLGFA

>HmALMT10

MAEKKEASSGLEWRIRVENGTSEILVPECDPSNKAWVGLHTLLVNLAMRLWKFFKKAWDI

GVEDPKKVIHCLKVGIALTVVSLFYYMRPLYEGVGGNAMWAVMTVVVVFEYTVGASLCKC

LNRICGTCLAGFLAIGIHWTASQSGEQFGPIIVGTSVFLLASAATFSRFIPVVKARFDYG

AMIFILTFSLVSVSGYRVEKLFDMAHNRMSTIIIGTSLCITVSMLVCPIWAGTELFLLIT

CNMDKLANSLDCCVAEYFIDNGGNTDSSEESDKKLQGYKCVLNSKATEESMAGFARWEPA

HGRFGFQHPWKQYLKIGESMRNYAYCIEALSSILNSEDQTPEVIKKHLHVICLRVSSNTS

NVIRELAMNMNTMRKSSKVDISVEEMNSSVQELQDRLKSLPSILIVAAASEPKNPEKERA

AITPIVEVIPLVTFASLLIEIAAKTEGIVVAVEELANLAEYKPEDDEKAQSKPAPITRL

>HmALMT11

MAADAEKTDQIHDNIKKMKRLEVVAQKVKKLPFLTFQIIWKVGREDPRRVIHSLKVGLSL

TLVSFLYLMEPLFKGIGQSAIWAVMTVVVVLEFTAGATLCKGLNRGFGTLLAGSLSFFIE

FIATESGRASRAIFIGAAVFLIGMASTYIRFFPYIKKNYDYGVVIFLLTFNLITVSSYRV

DNVLKIAHDRFYAIAIGCGICLLMSLLMFPNWSGEDLHNSTVSKLDGLAKSIEACVKGYF

SEEEKDQIAKYKSLEDPIYKGYKAVLDSKSKDETLALHASWEPRHSRHCYRFPWQQYVKL

GAVLRHFGYTVVALHGCLQTEIQTPRSVRALFKDPCIRLAREVSKVLIELANSIKNRRQC

STETLSDQLQDALQDLNTALKSQPRLFLSRKGTQTTTNMLAGLAASSAGQKSDVSLSSVK

TDTSALLEWRSKRASERSKEADRKVLRPTLSKIAITSLEFSEALPFAAFASLLVEIVARL

DLVIEEVEELGRIAKFKEFKDGDEITVNCESTSPKMGNVENHLPSHGTD
