# Supplementary material for: Identification of aluminum-activated malate transporters (ALMT) family genes in hydrangea and functional characterization of HmALMT5/9/11 under aluminum stress
Source: PeerJ. 2022 Jun 24;10:e13620. doi: 10.7717/peerj.13620 (PMC9235816; doi:10.7717/peerj.13620)
Supplement: Supplemental Information 3 [file peerj-10-13620-s003.docx]

>AtALMT1

MEKVREIVREGIRVGNEDPRRIIHAFKVGLALVLVSSFYYYQPFGPFTDYFGINAMWAVM

TVVVVFEFSVGATLGKGLNRGVATLVAGGLGIGAHQLARLSGATVEPILLVMLVFVQAAL

STFVRFFPWVKTKFDYGILIFILTFALISLSGFRDEEIMDLAESRLSTVVIGGVSCILIS

IFVCPVWAGQDLHSLLASNFDTLSHFLQDFGDEYFEAREKGDYKVVEKRKKNLERYKSVL

DSKSDEEALANYAEWEPPHGQFRFRHPWKQYVAVGALLRQCAYRIDALNSYINSDFQIPV

DIKKKLETPLRRMSSESGNSMKEMSISLKQMIKSSSSDIHVSNSQAACKSLSTLLKSGIL

NDVEPLQMISLMTTVSMLIDIVNLTEKISESVHELASAARFKNKMRPTVLYEKSDSGSIG

RAMPIDSHEDHHVVTVLHDVDNDRSNNVDDSRGGSSQDSCHHVAIKIVDDNSNHEKHEDG

EIHVHTLSNGHLQ

>AtALMT12

MSNKVHVGSLEMEEGLSKTKWMVLEPSEKIKKIPKRLWNVGKEDPRRVIHALKVGLSLTL

VSLLYLMEPLFKGIGSNAIWAVMTVVVVLEFSAGATLCKGLNRGLGTLIAGSLAFFIEFV

ANDSGKVLRAIFIGTAVFIIGAAATYIRFIPYIKKNYDYGVVIFLLTFNLITVSSYRVDS

VINIAHDRFYTIAVGCGICLFMSLLVFPIWSGEDLHKTTVGKLQGLSRSIEACVDEYFEE

KEKEKTDSKDRIYEGYQAVLDSKSTDETLALYANWEPRHTLRCHRFPCQQYVKVGAVLRQ

FGYTVVALHGCLQTEIQTPRSVRALFKDPCVRLAGEVCKALTELADSISNHRHCSPEILS

DHLHVALQDLNSAIKSQPKLFLGSNLHRHNNKHQNGSISNNKHHQRNSSNSGKDLNGDVS

LQNTETGTRKITETGSRQGQNGAVSLSSFRTDTSALMEYRRSFKNSNSEMSAAGERRMLR

PQLSKIAVMTSLEFSEALPFAAFASLLVEMVARLDNVIEEVEELGRIASFKEYDNKRDQT

ADDVRCENPANVTISVGAAE

>AtALMT9

MAAKQGSFRHGILEKRERLLSNNGFSDFRFTDIESNDLLENENCGRRTRLCCCCSCGNLS

EKISGVYDDAKDVARKAWEMGVSDPRKIVFSAKIGLALTIVALLIFYQEPNPDLSRYSVW

AILTVVVVFEFTIGATLSKGFNRALGTLSAGGLALGMAELSTLFGDWEEIFCTLSIFCIG

FLATFMKLYPSMKAYEYGFRVFLLTYCYILISGFRTGQFIEVAISRFLLIALGAGVSLGV

NMFIYPIWAGEDLHNLVVKNFMNVATSLEGCVNGYLRCLEYERIPSKILTYQASEDPVYK

GYRSAVESTSQEESLMSFAIWEPPHGPYKSFNYPWKNYVKLSGALKHCAFTVMALHGCIL

SEIQAPEERRQVFRQELQRVGVEGAKLLRELGEKVKKMEKLGPVDLLFEVHLAAEELQHK

IDKKSYLLVNSECWEIGNRATKESEPQELLSLEDSDPPENHAPPIYAFKSLSEAVLEIPP

SWGEKNHREALNHRPTFSKQVSWPARLVLPPHLETTNGASPLVETTKTYESASALSLATF

ASLLIEFVARLQNVVDAFKELSQKANFKEPEIVTTGTDVEFSGERVGLGQKIRRCFGM

>AtALMT4

MADQTREAFLSRKACSDFGFNDSNIIDDRRSKFRCFRFCSDGITASWKALYDIGAKLYEM

GRSDRRKVYFSVKMGMALALCSFVIYLKEPLRDASKYAVWAILTVVVVFEYSIGATLVKG

FNRAIGTLSAGGLALGIARLSVSAGEFEELIIIISIFIAGFSASYLKLYPAMKSYEYAFR

VFLLTYCIVLVSGNNSRDFFSTAYYRFLLILVGAGICLGVNIFILPIWAGEDLHKLVVKN

FKSVANSLEGCVNGYLQCVEYERIPSKILTYQASDDPLYSGYRSVVQSTSQEDSLLDFAV

WEPPHGPYKTFHHPWANYVKLSGAVRHCAFMVMAMHGCILSEIQAAPEKRQAFRQELQRV

GNEGAKVLRLFGEKVEKMEKLSPGNVLKDVQRAAEELQMKIDSNSFLLVNSESWAAMKEK

AEAEEAQQNYHEAKDDESKVIQSLSQIWDNNNNPHHQNQHAGNDSQLWISTESMMLRNRE

NWPSVSFIGGSMINEIESKVYESASSLSLATFASLLIEFVARLQNIVNAYEELSTKADFK

EQVSETRI

>AtALMT6

MGPFHQQSRERLLSQNEYSDMCFRKITNLCELGHSDRRRIFFAVKMGMALALCSVVIFLK

EPLHDASKYSVWGILTVVVVFEYSVGATLVKGFNRAIGTVSAGGLALGIARLSVLSRDFE

QTIIITCIFLAGFIASYSKLHPAMKPYEYAFRVFLLTFCIVLVSGNNTGDFFSTAYYRFL

FIVVGATTCLVVNIFIFPIWAGEDLHKLVANNFKSVANSLEGCVNGYLQCVEYERVPSKI

LTYQTSDDPLYSGYRSAIQSTNQEESLLDFAIWEPPHGPYRTFNHPWKNYVKLSGAVRHC

AFTVMAIHGCILSEIQAAPEKRQAFRHELQRVGNEGAKVLRLIGEKVEKMENLGPGEILN

DVQRAAEELQMKIDSKSYLLVNSESWAATKEKAEAEEYEEEAHETKVIKSLSQIWDTNSS

SNNQNPASGNDESQIWESTESMMLRNRETWPSVSFIGGSVVNETVYKVYESASSLSLATF

ASLLIEFVARLENLVNAFEELSTKADFRDPVPLNVVDQEGLWTTLMRLRRLRRYFGRS

>AtALMT14

MSDRVHERSMGMEEEGSTKNMKTKVLELPTKIKKILKNIWKVGKDDPRRVKHALKVGVSL

TLVSLLYLMEPLFKGIGNSAIWAVMTVVVVLEFSAGATLCKGLNRGLGTLIAGSLAFFIE

FVANDSGKIFRAIFIGAAVFIIGALITYLRFIPYIKKNYDYGMLIFLLTFNLITVSSYRV

DTVIKIAHERFYTIAMGVGICLLMSLLVFPIWSGEDLHKSTVAKLQGLSYSIEACVNEYF

EEEEKDEETSDLSEDTIYNGYKTVLDSKSADEALAMYASWEPRHTRHCHRFPWKHYVKVG

SVLRQFGYTVVALHGCLKTEIQTPRPLRGLFKDPCVRLAGEICKVLSELAASIRNRRHCS

PEILSDSLQVALQDLNTAIKSQPKLFLGSSQNGNVSQGNSGRHNPNVAVSQHINKDTNEA

ASYQNTGTPRGERMSRFGPNVSFSRLRADTLERRSAAATNERKILRQQLSRIVVLTSLEF

SEALPFAAFASLLVEMVARLDNVIEEVEELGTIACFKDYDNNVDQKDVEVRVEKPADLVV

GVE

>AtALMT3

MAAPKLESFRRGSMFDGSFRRGSMFDGSFRQSMRDRLILQSRGYSNVNDDDKTSVRCCSY

SYFSDKITGVVKKLKDVLVTAWEMGTADPRKMIFSAKMGLALTLTSILIFFKIPGLELSG

HYLWAILTVVVIFEFSIGATFSKGCNRGLGTLSAGGLALGMSWISEMTGNWADVFNAASI

FVVAFFATYAKLYPTMKPYEYGFRVFLLTYCYVIVSGYKTGEFMETAVSRFLLIALGASV

GLIVNTCIYPIWAGEDLHNLVAKNFVNVATSLEGCVNGYLECVAYDTIPSRILVYEAVAE

DPVYSGYRSAVQSTSQEDTLMSFASWEPPHGPYKSFRYPWALYVKVGGALRHCAIMVMAL

HGCILSEIQAAEDRRREFRNELQRVGIEGAKVLRYIGESLKKMEKLNPIEDILYEIHQAA

EELQSKIDKKSYLLVNAKNWEIGNRPRVRDLTDEQKISNLDSDLSRILAHKSQSEATLRP

PKNWDDVTTAANLSSATMLPYLQSRTMIHKQPSWPSRISITPGSMLQPPLGEPGKMYESA

SNLSLATFASLLIEFVARLENLVNAYDELSVKANFKEAVSE

>AtALMT5

MGGKMGSVPEQNTEKLLWQSSDVADSRDSKFRCCSWRALYEAPAKLYALGHSDRRKLYFS

IKMGIALALCSFVIFLKEPLQDASKFAVWAILTVVLIFEYYVGATLVKGFNRALGTMLAG

GLALGVAQLSVLAGEFEEVIIVICIFLAGFGASYLKLYASMKPYEYAFRVFKLTYCIVLV

SGNNSRDFLSTAYYRILLIGLGATICLLVNVFLFPIWAGEDLHKLVAKNFKNVANSLEGC

VNGYLQCVEYERIPSKILTYQASDDPLYSGYRSAVQSTSQEDSLLDFAIWEPPHGPYKTF

NHPWKNYVKLSGAVRHCAFTVMAMHGCILSEIQASPEKRHVFSNELRRVGNEGAKVLRLF

GEKVEKMEKLSLSLGEILKDVQRAAEALQMKIDSKSYLLVNSESWAAIKEQAEAEEAREN

DQEAKDDETKVIKSLSQIWDTNNNNNHQSNDQSQHWMSTESMMLKNREMWPSMSFIDGTV

VNEIECKVYESASSLSLATFASLLIEFVARLQNIVNAFEELSTKAGFKDAVDQIPKV

>AtALMT2

MEKVREIVREGRRVGKEDPRRVVHAFKVGLALALVSSFYYYQPLYDNFGVNAMWAVMTVV

VVFEFSVGATLGKGLNRAVATLVAGGLGIGAHHLASLSGPTVEPILLAIFVFVLAALSTF

VRFFPRVKARYDYGVLIFILTFALISVSGFREDEILDLAHKRLSTVIMGGVSCVLISIFV

CPVWAGQDLHSLLASNFDTLSHFLQEFGDEYFEATEDGDIKEVEKRRRNLERYKSVLNSK

SNEEALANFAKWEPRHGQFRFRHPWRQYLAVGALLRQSAYRIDALNSNINSDMQIPMDIK

KKIEEPLRRMSSESGKSMKEVSISLKNMTISSSFDIHVVNSQSACKTLSTLLKSGILNDV

EPLQMISLMTTVSLLIDIVNLTEKISESVHELASAAKFKNKKKPSKSNSGSIGQAMPNKS

HDDDDHVVTILGDVDTSNNVDQSQSHGEISVDSCHHVTIKINDDDSIHDKNEDGDIHVHT

NRVSCDHTNASDLLDSGVKKN

>AtALMT13

MGYKVEARSMEISMEDEDSRKKRKKGLNLPKKMKKILRNLWNVGKEDPRRVIHALKVGVA

LTLVSLLYLMEPFFEGVGKNALWAVMTVVVVLEFSAGATLRKGLNRGLGTLIAGSLAFFI

EWVAIHSGKILGGIFIGTSVFTIGSMITYMRFIPYIKKNYDYGMLVFLLTFNLITVSSYR

VDTVIKIAHERLYTIGMGIGICLFMSLLFFPIWSGDDLHKSTITKLQGLSRCIEACVSEY

FEEKLKDNETSDSESDDEDLIYNGYNTVLDSKSADEALAMYAKWEPRHTRRCNKFPSQQY

IKVGSVLRKFGYTVVALHGCLQTEIQTPRSIRVLFKDPCVRLAGEICKVLSELSESIQNR

RHCSSEILSDSLEAALKDLNSTIKSQPKLFLGSNLHSNITNKHLNGHVSYYNETNSNGTV

SYHNDNNTNGCVLGETIEENDTVSPLPLNSVVSLSSLRSVKKSAATGEKRRLRKQLSKIA

VMKSLEFSEALPFAAFASLLVEMVARLDTVIDEVEELGTIACFKEYDKTVEVRIENRLI

>AtALMT10

MATQEAGKLEWRISVDNGTTERLVPRSGLSKRIFLWLKDLVMKVIMERVAKFMRKAWRIG

ADDPAKVVHCLKVGLALSLVSIFYYMRPLYDGVGGNAMWAIMTVVVVFESNVGATFCKCV

NRVVATILAGSLGIAVHWVATQSGKAEVFVIGCSVFLFAFAATYSRFVPSFKARFDYGAM

IFILTFSLVSVGGYRVDKLVELAQQRVSTIAIGTSICIIITVFFCPIWAGSQLHRLIERN

LEKLADSLDGCVAEYFKENEVSTNRNEDENTNMKLQGFKCVLNSKGTEEAMPLIRFSGFS

FSQANLARWEPAHGSFNFRHPWKLYVKIGAAMRRCAYCLENLSICINYETEAPDQVKNHF

GEACMKLSSASSKILRELADMMKNTRKSSKMDFLVFDMNSAVQELQETLKNVPIETNKPE

EVPSEEENKVDSEERTTSMSLHEVLPVATLVSLLIENAARIQTAVEAVDELANLADFEQD

SKKKTGDNNTKQPPLSS

>AtALMT11

MSNKVHVGNIEMEEGLSKTKWMVLEPSEKIKKIPKRLWSVGKEDPRRVIHAFKVGHSLTL

VSLLYFMENLFKGIGSNAIWAVMTVVAVLLEFFAVEGLTISEKVILSMAARGRESAAEPH

ERNEAGNVCHSIKFLPKSIARAKQHHVLNQPY

>AtALMT7

MEKVREIVREGRRVAKEDPRRVVHSFKVGLVLALVSSFYYYQPLYDSFGVNAMWAVMTVV

VVFEFSVGATLGKGLNRVAATLFAGGLGIGAHHLASMSGPTGEPILLAVFVFVQAALSTF

VRFFPRVKARYDYSLLIFILTFALISVSGFREEQVVKLTHKRISTVIIGGLSCVIISIFV

CPVWAGQDLHSLIASNFEKLSFFLLGNSFHYVSSDLNSITLLRKIKSWRLADFGDKYCEV

VENDGAKEVDKRKKDFDNYKSVLNSKSNEESLANFAKWEPGHGQFRFRHPWKQYLAVGEL

IRQCAYRIHALNSYLNADNQVSVDIKKKLGEPLRRMSLESGKAMKEMSISLKKMTKPSSS

DLHVQNAKSACKSLTNLLNSGILKEVEPLELVSLLTAISLLIDIINLTEKILESLHELAT

AAKFKNKIEHPLFSEKPKAKSFVSVRSIKCHDDHVVIIIEDDGNNDDTSKNDNGSKEVSI

HEKHEDDDTHVDARCVSCGHTSVCVK

>AtALMT8

MDLNAQEKKAGFFQRLQDFPSKLKDDVTKRVKNVQKFAKDDPRRIIHSMKVGVALTLVSL

LYYVRPLYISFGVTGMWAILTVVVVFEFTVGGTLSKGLNRGFATLIAGALGVGAVHLARF

FGHQGEPIVLGILVFSLGAAATFSRFFPRIKQRYDYGALIFILTFSFVAISGYRTDEILI

MAYQRLSTILIGGTICILVSIFICPVWAGEDLHKMIANNINKLAKYLEGFEGEYFQPEKI

SKETSSCVREYKSILTSKSTEDSLANLARWEPGHGRFRLRHPWKKYLKIAGLVRQCAVHL

EILNGYVLSNDKAPQEFESKIQEPITTMSREVGEALKAIAKSIKTMRNDSACVNAHIDNS

KKAIKNLKIALKSSYPETYKDLLEIIPGVTMASILIEVVNCVEKIYEAVEEFSGLAHFKE

TLDSKLSAEIGQHQLLHRGCVKPVLDGDNEKEDNSSCHVLITVHDEGYLPTATAKNVLGA

EKTRVDIV

>PKA63368.1

MNGKKGSIKVDIPKSEVANNPLNIDVGSPATSHWVREVWEFVREDTDRVIFSLKAGLAVLLVSLLILIRA

PYNVFGTSIIWSILTVALMFEYTVGATFNRGFNRALGSLFAGILAVLVMQVAMACGQIVEPYIIGFSIFI

IGAATSFMKQLPSLAPYEYGFRVTLFTYCLIIVSGYRMGSPARTAMNRLYSIAIGGLVAVFVNALVFPIW

SGEQLHRELVGSLHAVANSLEECVKKYLADDGSSLPEFSKTVADDFPDEPAFRKCRKMLNSSAKFETLAS

SAKWEPPHGRFRQFFYPWPEYVKVGAVLRYCAYEVMALHGCLHSEIQAPYNLRCTFQPEILDAANQAAEL

LRSLAKDINSMKWSLHGNLLKRVHSSTERLQRSIDMHSYLLTSTHEIHDCPAKLPESQDMSDQPKESESK

NEQNNLSNAKLEQAESYHEAMRKQQRRLYSWPSREVDDFEDEDVDNKIIQKMRAFESTAALSLATFTSLL

IEFVARLDHLVDATDVLAKMAKFKKEYTS

>PKA62927.1

MLEVVHDRLGDNAMWAVMTVVVVFEFTAGATVSKGMNRGAGTVVGGGLGSIVATLAQEIGGLGKALAIGI

SIFIFGAAATYMRMIPNIKNKYDYGVMIFILTLSLVAISGVRGDEIIQVASDRLTSICMGLAVCAFMSFF

IFPVWAGDELHSSLASKFTSLAQSIQDCLEDYVNQTNGKKGAEERRISACLSVLSSKTNDESLANFAKWE

PWHGRFGFYYPWNKYLQIGGILRELAACVISAKGRLKQKQEA

>PKA62717.1

MGPPPPRLGSFRYHFEQRSKERLPLLSSLKDVDWGNFDLEDDGGGRRCFRRARDGISWIWGRVAAMAVAG

WEMARSDPRNLVFAAKMGLALAVISLLIFLKEPLPDLSSHSVWAILTVVVVFEFSIGKLLPFSSPSSIGA

FDLMLRVDSGLVSSDIDDNLYRLGGSTQLSGEKLPGLSQVLGRYLNRVEYERVPSKILTYQASDDPLYSG

YRAAVESKAQEDALFGFAIWEPPHGRYKMFKYPWKSYVKVSGALRHCAFMVMTLHGCILSEIQVKCFLVL

FKSIDSTFRVDRGRLDVPIAPSFVLDLGYRTMAPLERRQVFRSELQRVGIEGTKVLQELGHRVKTMTKIN

SSEILLEVHDAAEELQRKIDRRSYLLVNSELWEIGRRPEVGVEAVDPINTVDTRPVQIGIKSPSEAVLDN

RSLHLSRSWDVHSAGTGCEPILKQQISWPVRPSFSIDDAPLEEESRTYESASALSLATFSSLLIEFVARL

QNVVNAFEELSEEAKFKEPADEPGTDSAPGLWTRIKKFFGF

>PKA60074.1

MEEKVEKLMKSLKKLPVSVCRKVWRVGRDDPRRIIHALKVGLALTLVSFLYLLEPLFEGVGQNAMWAVIT

VVVVLEFTAGATLCKGLNRGFGTLCAGSLAFFIEFMAEQSGKVFRAIFIGTSVFLIGFLSTYLRFFPYIK

KNFDYGVVIFLLTFNLITVSSYRVQNVIHIARERLYTIAIGCGICLFMSLLVFPNWSGEDLHNSTVKKFE

ALARSIEGCINEYFQEKDEQESKDKVEDPIYRGYRAVLDSKSSDETLATFASWEPRHNRHCHKDPWKQYV

KLGAVLRHFGYTAVALHGCLQSEIQAPKSVRALFRDPCIRVASEVSKVLVELADSIRNRRHFSHDVLSDH

LQEALQDLNSAIKSQPRLFLGSKNNRTKFIGPKHEKEPLFDVAMPSLKTDTSALLEWKTNRVTDQPKEPA

EQKQLRPTLSKIAITSLEFSEALPFAAFASLLVEMVARLELVIEEVQKLGRAAQFREFTCKDEIAIKVND

ENRKHVETSHDLNTHEVHQIVD

>PKA56322.1

MAATDRRETTVASIVTGEWRVTIPKESLTTVKHEHGLVRRAWLATIAKVAAGEKMVAKIASDDPRKVVHA

FKVGITLALVSLFYYLRPLYDGVGGTAMWAVMTVVVIFEYTAGGTLYKGLNRAAATLTAGALGVGIHWFA

SKAGHAGELIILNGAVFILASVVSFSRFFPAIKRQFDYGATIFILTFCLVAVSGYRVDELVVLAQERLST

VCIGIAICLIVSMLICPVWAGGDLHRLTAGNADKLADSLEGSVEGYFDGKEAPKAQGFKCVLNSKALEDS

LVNLARWEPGHGPFSFRHPWSQYKRLGDAMRNCAFCIEALYGCLTSEIQAPELMKKRLSDACLKLSSNSA

KALRETAACIRLMERSKTLECLIGETSCAAAEVKRELPELLPAGDAEVDHQLVTTKRNHSLIEAMPLVAF

ASILAEIPARVKGVAEELEKLADLAGFKAAGERLSN

>PKA53938.1

MEAPAGTSEKVGFSPWSKLRETAIGAGRKLRKIGTDDPRRILHSLKVGLALTLISIFYYVKPLFHGLGAS

TMWAVLTVVVVMEYTVGGTLSKGLNRAFATLLAGALGLGAHQLAALCGDKGEPILLSVFVFLLAAMATFS

RFVPEIKARYDYGVTIFILTFSLVAVSGYRVDQLLQLAHQRLSTIAIGVATCLATSIFVFPVWAGEDLHD

LVALNLEKLANYLEGLGKEHFGEKMETKKMVDLTFLQSYKSVLNSKSIEDALANFARWEPGHGGFGYRHP

WKHYLKIGALIRQCACSIEALNAYISIAENDQMTVTDEEFLQRIRADCAAMSSESGKALAELAAGIRQME

CPLAAVQHMSRAAAAAGGVKTAAPAEDASMPEVLRSATTASLLSEVVRRASHVVAAVEELSQLACFKRPE

NSHKAGAINPVAGVESPPHVVVYIEK

>PKA47830.1

MKHKLWGFAYLLWSTGKSDPRKGIHALKTALALTLVSIFYYVRPLYQGVAGSSMLSITTVAVVFEFDVGG

TIYRSFNRVSATIIAGFLAIGIHIIADRSTKEVGHIVHGTTVFLIALAATFARFIPAVKSRYDYGMLIFI

CTFTLITLSGHQIGKLLEIAIRRSLTIALGSSICVLVSMVIYPIWAGEELQLLLAGNLHKLASSLEVFMV

EYFEKGDHELKNKESIQKLISGYRCVLSSKASEDRLASLARWEPPHGRFGIKHPWKSYKKVGVAIRYSAS

CIETLHSYLKFEENIRAPESIRNHLSSAFMRLCFCSSSTLKELMNSMKEMKKSSTMGDLVANMNYAEEEL

QLALKTLPNLIIRLKLYKTSNINNSGMENMSSRFVEVVPLVTVSSLLMEISTRIKSINDEFNDLARDIFT

DEIKEREEVC

>XP_012843873.1

MAANLGSLRQSFVERGRERLLSRKYNSDVGFNDSSYIVHEGCLHWLFRVTVDRISRWWNNVTGIAFSAYE

MGRSDPRKVVFAAKMGAALSLVSVLIFFKEPLSYISQYSIWAILTVVVVFEFSIGATLNKGFNRALGTLS

AGALALGIAELSKMAGEMQELFIVISIFIAGFFASYLKLYPAMKQYEYGFRVFLLTFCIVLVSGTSHFVE

TAVSRLLLIAVGAVVCLLINVCVYPIWAGEDLHKLVVKNFKGVATSLEGCVNMYLQCVEYTRIPSKILLY

QASDDPLYKGYRAAVESTSQEESLLGFAIWEPPHGRYKMFNYPWSEYVKVSGALRHCAFMVMAMHGSILS

EIQASSELRQVFKDRIQRVGTEGAKVLRLLGEKVEKMEKLSPSDLLEEVHNAAEDLQMLIDQKSYLLVNA

QSWESQKRPENNPDPELLQELKDNEHKPPPLMINSLSESVNFKSAQTLSRNYDPHNPNTSINMSVSQQWG

SAEDVLRQQTMWPSRLSLIGENILNEREVRTYESASALSLATFTSLLIEFVARLQNLVHSFEELGDKAKF

TDPIDSNESKEVVDLLA

>XP_012833218.1

MSKEKLLLFHRFNDEKSSFHSLRVRVVQLWSDYWGFFKKALEMGRKDPRKIIYAVKNGFALAVVSLLIFW

KDPLSDVSQFAIWAILTVIVMFEFSIGATFIKGFNRGLGTFCAGMLVFLFAELAILAGDCEKVVIVISFF

IIGSIASYLKLYPTMAPYEYGFRVFVLTYCILMVAGNRTREYTTAILTRLVLIAVGACICFIINISIYPI

WSGDDLHSLVVNNFKDLATSLEGCVNGYLKYVEYERAPTRFSTQATEDQLYYKSVIESATKEKTLLGFAV

WEPPHGRYKKLGHPWTTYVKVSTAVRHCAYTVMALHGCILSEIQAPPEKRQIFRSQLQSVSAEGARVLRQ

LGDKIEKMEKIGTSENILKQVHEAGEQLQKKIDQRSFLLVNSGNWEIGNPTNTELDYQDDDVVSIERGQQ

HDSLQLGFKSQSEAAIHIPSSLTFVKSLANKDLKKKPQTLRKLVPWPSWISFEGAGLIKEDEVKTYQSAS

ALSLATFASLLIEFVARLQNVVDCFEELSEEAGFADPDVVLLEERRFGHWARLCTFRFKA

>XP_012834168.1

MYSGIAMRIAGESPCWDWTDKTDIIKKRSIFFVQKFKKLAISTWMTICRVGQEDSSRVFHSLKVGFSLTL

VSLLYLLEPLFQGIGQNAIWAVMTVVVVLEFTAGATLCKGLNRGIGTLLAGSLAFLIQYIAEESSHIFRA

IFIGTAVFVIGSAATYMRFFPYIKKNYEYGVVIFLLTFNLIIVSSYRIHNVLKIAQERFYMIAIGCGICL

LMSLIVFPNWSGQDLHHSTISKLEGLAFSIEACVNEYFSNEDRKAAKDTTLEDPIYKGYKAVLDSKSSDE

TLAMHASWEPCHSRYCYRLPWQHYVKLGEVLRRFGYTVVALHGCLQTQIQTPRSVRALFKDPCIRVAGEV

SKALIELSESIQNRRQCSPEIISDHLHEALQDLDAALKSQPRLFLGPNTATKNMLTFAAVTHKSSTTTRG

GGVSLASVKTDSSALLQWKSKRAAKEQANNNDSLRPTLSKIAMITSLEFSEALPFAAFASLLVEAVARLD

LVIDEVEELGRVANFREFNTCDADQVTKTCTKPTTPLHNHLPSHGVD

>XP_012835628.1

MVVKESEVCGGGLEWRINFPDGTSKILVPHHHTISKTRFCRILHAFFVEGLFSKIYGFFREAWRLGMDDP

KKFFHCFKVGIALSLVSLFYYMRPLYQGVGGNAMWAVLTVVVVFEYTVGATLCKCLNKATATFLAGALGI

GVHWIGSHFGEKLQPIFLQGSVFVLAAAATFTRFMPSVKARFDYGVLIFILTFSLVSVSGFRVGKLFEFA

SHRLSTIAIGTSICILTSILFCPVWAGEELHNLIQTNMEKLADSLDECVAEYFRKDARSEASNKISVGYR

CVLNSRATEESLANFARWEPAHGGFNFGHPWKEYRKVGASLRSCAYHIEVLNGSINAGTKAPDFLKKHFS

KFCMKLSSSSSAVLKELVFVTHTMTRSAKIDIIVEEMRSAVEELQIVLKSLITQPINASTNINIGNEESD

ASNTSMSLIEIAPLVTLSSLLIEIAARTEDIAKAVNELAN

>XP_012835823.1

MKSIARKIKSIFMETVKETKRVGEEDPRRIIHAMKVGFALTLVSLFYYFRPLYNGFGQAGMWAILTVVVV

FEFTVGGTLSKSLNRGCATLVAGALGVGAEYLAALCGDKGEPVVLGLMVFFLATTSTFTRFFPNVKRKYD

YGVLIFILTFSLVAVSGFRVDQILHLAHRRLSTILIGGASCIVISIFVCPVWAGQDLHNLIAANIEKLGA

FLEGFGTEFLIFHGDESGKINSSCKHGDQKSFLEGYKSVLNSKATEESLANFAWWEPPHGRFRFNHPWKQ

YLKIGALARDCACLIQSLNGYINSKSQSLVQVKMKIKESCMKMSMESGKALKELASAVKTTAFHPLLAAG

AHLQNSKSAADDLKIALENSSLLSAKADLQEIMPLLVVASVLIDINDGVDKIYESVNELSLKACFKNNPK

SSKACELHQQVIIHRGVVKPVNDSEEHVVVVVVDGGASAEESPEKENRHPPPQ

>XP_012838150.1

MQIESENLDKKVGFFLSGWLWIKGLIEKFFAKLREIAKQIEKTAKDDPRRLIHSLKVGVALTLVSLFYYC

QPLYRSFGVSSMWAVMTVVVVFEFSVVFFFEGATLGKGLNRGMATLVAGGLGIGAHYLAMVAGKTCEPIL

IGLFVFLQAVASTFIRFFPKVKARYDYGMLIFILTFCLVAISGLRSDEIIEIARKRVLTILVGALTCVVV

SVFIYPVWAGEDLHLLVAQHLDKLGHFLEGFGERCLRTIEESDNLVGLNTVLDSKSREEILANFARWEPG

HGKFMYRHPWKQYLKIASLTRQCASRVDSLNAYLNSKSHAPKEIQGIISSMSRESGKALRELSLAVETMT

RPSPYPNCHVSSLKAASKNLNSLLKSELWGEHANLLQVIPVAAVASLLDDTVTSVEKIADAVDELSSLAN

FGNAIGGETTEDKSRNGVVTLVGIVIEGSDQVKKGQSSGLQAG

>XP_012838155.1

MASQENQENVRNVKSAWSRLKGFPDKCVDLVTTSAREAKNFGKEDPRRIVHSFKVGLAITIVSLFYYFDF

LYDGLGVSAMWAVITVVVVFEFSVGATFGRGINRAIATFIGGLLGVAAHRLASSTGNNAEPIVLGFSVFF

ITSIATFIRLFPKMKERHDYGCMIFILTFCLISVSGYRDSEVIEMAHTRLSTVMIGLSATGLICIFICPV

WAGEDLHNLTAANIEKLGISLEAFQRKYFETTRNDKNQENKPPVDGYKVVISSKSKEESLVNFARWEPRH

GKFKYRHPWDQYIKIAGHTRECAHRFYALYGYLHSEIPTPPIEISKKIEELCTTMSLECSHALKELAIVI

KKMTRSPNSEAHLKNAEIAAKNLKLLVQSGLWPESDPLDVIPAAAVALLLIEIVCSTVKIADSVHRLSIL

SKFKSVDPARTPARFGVTVE

>XP_012838156.1

MASEQNQEKFRGLKSAWSFLKGFPGGIVNAVVSVAREAKKLGKDDSRRIVHSFKVGLAITIVSLFYYFDF

LYDGFGVSAMWAVITVVVVFEFSVGATIGKGVNRGIATLVGGVLGVAVHCLASVTGKHAESIVLGLSVFL

ISTAATFIRFFPQLKARYDYGLLIFILTFCLISVSGYRDDEVIEMAHTRLSTVAIGSCATVLICIFICPV

WAGEDLHNFTADNIETLGIFLEDFQGKYFETTNDKSEENEENQDSLDGYKVVLNSKGTEDSLINFAKWEP

RHGKFKYRHPWDQYVKIGKSTRECAYKIDALNCYVISEMHR

>XP_012842921.1

MASDNNQESSVLKELPKIAWTRLKGCSDKVVSTVMCPVRDAKNLGKQDPRRITHSCKVGLAVTLVSLLYY

FDFLYDGFGVSAMWAVMTVIIIFDFTVGATLGKGVNRGVATLIGGALGLAAHRLTSFNGRIVEFSVLGFF

IFLISALATFIRFFPKVQARYDYGLLVFILSFCLISVSGYRNDEVMLMAYRRLSTVMIGGVLTVFISIFV

CPIWAGEDLHNLTANNIEKLGIFMEGFERRYFETNNDKKQEKKASPDGYITVINSKSTADTLVNVAKWEP

RHGRFKYWHPWEQYLKIGANTRECACKIDALNCYLNSKIQTPMEIREKIQEPCTTISQQCSHALRELSVG

IKKMSSPAMLSDPHIKNAEAAAKSLESLLQNGKWLEIDFRDMIPAAAVALLLIEMVSCTAKLADSVHELA

SMSKFRAPYDNAVKANQTCLERVVVVTRVSNDHKVSLLC

>XP_012843961.1

MVKESGGLEWRINVSDGTSKVLVSESTSQSRVCRGLMRFFSKISDFFRKAWSMGVNEPKKTMHCVKVGMA

LTLVSLFYYMRPLYEGVGGNAMWAVMTVVVVFEYTVGATLSKCVNRAIGTFLAGALGVGVHWIASQSGEK

FEPIILQCSVFLLAAAATFSRFIPSIKARFDYGAMIFILTFSLVSVSGYRVEKLFELAHNRLSTIAIGTS

ICILTSMLIYPVWAGTELHNLVKNNMEKLSDSLEGCLAEYFKDADSGDCNKKMQGYKCALNSKATEEAMA

NFARWEPAHGSFNLGHPWKEYIKVGALIRSCAYCIETLNGSISSDTKFRELIFFVQTLQVPDFLKKHFRK

FCIRLSANSSAVLKELATSIGTMTRSSKVEFLVEEMRSSVQELQNVLESLSKQPIQSTAPKNEGGTAEGN

SSPIVVTLVQIVPLVTVSSLLMEIAARTEKIADAVNVLAEKAEFRSEKDEKPKKGQNPKAVDRNSGESEE

DKTMMTLQKV

>XP_012847503.1

MEMNNTIEYSEKQEKIGSAICERVNNFFKGSYEKFDKSKVTEIAKIEDEDRRRIIHSVKVGLAITLVSFL

YYLRPLYDGLFGQAGMSAVLTVVVVFEFSSVGALFSKCLNSGAATLVAGGLGVGAGYLARLCGDKGEAIV

LGILVFLLAAASTYMRFIPSIKSKYDHGVVIFILTFTLVAILGYRVTEISILGHQRLSTIFMGGAICILV

SVSVCPVWAGQDLHLLVSGNIDKLATFFLGYGDELSTDKIDKYLEDHKTVLDSKVNEESLANFAWWEPPH

GEFRYNHPWEHYLELGVLTRECANHIQTLISTGYCINSKPQELESEFDLKIEPECKKLSTELGKALKELA

SATKGMTVPQLSAAQIHISNSKSAADELIIVLQNCSSLLLGPTKIDLGRIMPPLVTTSILVDVIECIERI

SASVDELSQKARFRKLDSSKDQKKLDDLHVVIDIKDELDAKQETL

>XP_012847931.1

MECQVWSWLISFVICTAKDAKKLGEEDPRRVIHSFKVGLAITLVSLFYYFDFSYQGFGVNAMWAVMTVVV

VFEFSVGATLGKGVNRAIATLLGGGLGVAAHRLASFAGEKIECVILGMSVFFIASAVTFCRFFPKMKARF

DYGLLIFILTFSLITVSGYRDDEVIDMAQRRLSTILIGGCATVFICMLICPVWAGEDLHKYTASSIEKLS

IFLEGFGRAYFQVSNEKNQENNVASLDQYKTVLNSKGIEDSLVNFAKWEPRHGKFRYRQPWDQYLKIGSL

SRECAYKIDAINSYLNSDAQTHVEIQKKIQPPCTKMSTECSYALRELAMGIKTMTCSLSADIHIVNAKAS

AKKLKSMLKNGLRPEIDLLEIIPTFTVASLLIEVVSCTVKIADCVHELGSKSKFKSPDPVKMKKQTSCKK

LTRSPSIERSHSFSISIE

>XP_012854166.1

MNGKKGSVEINIPPIAKAKQSETTKKCEEKFGTSFISSILNVWEFCKEDTNRVIFSLKVGLAVLLVSLLI

LCQSPYRVFGTNIIWSILTVAIMFEYTVGATFNRGFNRALGSLLAGIIAISIAELALKTGRIAEPIIIGF

SIFIVGAITSFMKLWPSLVPYEYGFRVILFTYCLIIVSGYRMGNPIRTAMDRLYSIAIGAIVAVVVNVLI

CPIWAGEQLHKELVNNFSSVADALEECVGKYLVDDGSEHSEFTKTVMDDFPEEPAYRKCRSTLNSSAKLE

SLANSAKWEPPHGRFRHFFYPWTEYVKVGAVLRYCTYEVMALHGVLHSEIQAPYNLRIAFRNEIREVSTD

AAEAIRRLGKDIRNMQRTLKISVLKKVHTSTEKLQRAMDMYSYLLTSSANHEPPHKQPLSHALSSDLDKK

LPEQNHDPPLLLQEEYSYHETMRKQSKRLYSWPSREVDAFEEGGLGFSSTDCMPRMKTLETTATLSVATF

TSLLVEFVARLDHLVEAVDELSKMAKFKQEDV

>XP_012857503.1

MATDNQENSIGLKQVWSWLISFVICTAKDAKKLGEEDPRRVIHSFKVGLAITLVSLFYYFDFSYQGFGVN

AMWAVMTVVVVFEFSVGATLGKGVNRAIATLLGGGLGVAAHRLASFAGEKIECVILGMSVFFIASAVTFC

RFFPKMKARFDYGLLIFILTFCLITVSGYRDDEVIDMAQRRLSTILIGGCATVFICMLICPVWAGEDLHK

YTASSIEKLSIFLEGFGRAYFQVSNEKNQENNVASLDQYKTVLNSKGIEDSLVNFAKWEPRHGKFRYRQP

WDQYLKIGSLSRECAYKIDAINSYLNSDAQTPVEIQKKIQEPCTKMSTECSYALRELAMGIKTMTCSLSA

DIHIVNAKASAKKLKSMLKNGRPEIDLLEIIPTFTVASLLIEVVSCTVKIADCVHELGSKSKFKSPDPVK

MKKQTSCKKLTRSPSIERSHSFSISIE

>XP_012858652.1

MAAANYGSLTQSFLDKTKDRGGAVSRKHFSDSIFEDSYFVRGNEGCLRRIYRSIGDKFSNWWGNIKATAI

SAYEMGRSDPRKVVFAAKMGSALSLVSVLIFFKEPSTYITKHSIWAILTVVVVFEFSIGATLSKGSNRAL

GTLSAGGLALVIAALSNMAGRFKEVMVVINIFIAGFLASYLKLHPAMKQYEYGFRVFLLTFCIVLVSESD

FTQTAVSRLVLIAVGAGVCLVMNVCIFPIWAGEDLHKLVVKNFRGVATSLEGCINMYLESIEYSRIPSKI

LIYQASDDPLYNGYRAAIESTSQEEALLSFAVWEPPHGRYKMFNYPWSEYVKVSGALRHCAFMVMAMHGS

ILSEIQASSELRQFFKDGIQRVGSEGAKVLRLLGEKVEKMEKLNPGDLLQDIHEAAENLQMIIDQKSYLL

VNAESWAGINAKRPENVPDQHHVQELKDNEHKPPFLIKSLSSINQQPGPPPSLRNYDARMANRSVTQSIS

EWGSGEDLLRQQTMWPSRLSLIGDAIFNEREARTYESASALSLATFTSLLIEFVARLQNLVSSFEDLGER

AKFADPTAGFAEVATGLGFWARLVRRVKG

>XP_012828642.1

FDLQEASSSELHLKIQPSCKKLSTESGEALRELATALKNKTFPSAAAETHIKNSKSAAEELKRVLENSSL

LPTKLDLQEIMPLLVTSSVLIDVVACVERIAVSVNELSEKAGFVREAVSADQKQLLRRGIVKPVDDEHGV

VIEINDKLGDSVENMKTVNII

>XP_012828644.1

MPIKLIKSKVMGTVKKTKKIGEDDPRRIVHSVKVGLALTLASLFYYLRPLYDGFGQAGMWAILTVVVVFE

FTVGGTLSKCINRGVATLLAGALGLGAEYLADLCGDKGEPIVLGLFVFLLAAASTFTRFIPHIKTKYDYG

VLIFILTFSLVAVSGYRVTQILQLAHQRLSTILMGGAMCVIISIFVCPVWAGQDLHNLVAGNIGKLATFL

EGFGGESFTSQGDDGDKDKLFLHLHKSVLNSKATEESLANLAQWEPCHGRFIFRHPWKQYLKIGTFSREC

ACLIETLTGCINSKPQEAAPSEIELQIQPAFRKLSTESGKALKELASAIKNMTFPSSAVIENHLNNSKSA

AEDLKTMLDNFSLSPNGKPDIQEIMTLLVIASLLTDIINCVEKISISVNELSEKARFRKENSTDNKQQRL

IHCGVVNPVDDDERTVVIEIKETTLGDLILKDKH

>XP_012828704.1

MEIIGSANQKKAGGVQRSVINFFTAMSEKLMENVKKTKKIGEDDPRRIVHSIKVGLALTLVSLFYYFRPL

YDGFGQAGMWAILTVVVVFEFTIGGTLSKSINRGVATLLAGALGVGAEYLAGLCGDKGEPIVLGLLVFLL

AATSTFTRFIPHVKKKYDYGVLIFILTFSLVAVSGYRVNLILQLAHQRLATILLGGATCIIISIFVCPVW

AGQDLHNLVSGNIEKLATFLEGFGGDDDKDKSYLEGYKNVLNSKATEESLANFAWWEPAHGGFRYNHPWK

HYLKIGGLSRECASHIETLAGYSVNSKHTQAKV

>XP_016444055.1

MEIDSTSKTDSKSKEQPGVFTRWWSQLKGFPRMLKDKSWNIAKDTKQIGKDDPRKIWHAAKVGLALTLVL

LFYYSWPLYHSFEQSAIVACLTVMVAFEYTAGATMSKCINIAFATALGGTLGIGAKYLAELCGKEGEPIV

LGFLVFILGALGTFTRFYPQMQRRYDYGCMLFVATFSLVTVSGDKYLDLDKQRISTIMVSVATVMIISLL

ICPVWAGEDLHNLVTTNLEKLASFLEGFGSEYFDVSEAEASGEGYKDNEKGFFEAFKSVLGSKATEESLA

NIAWWEPAHGSFRFNHPWKQYLKIGGIARECAGHLQSLSGHLKSKSQAPIEFNRRTEKACKRMITESSKA

LKELAFSIKIVTKLSTITTESHSYNARTAIADLKEALFTFKTFFLFEEADTIDVIGAMSVVSILIEVTKC

VDKISEAVEELSIKARFNKEENNEKKKKKKKKNDSSSAKALEKPPPRPQLLIPVLEADNVNSGASVVIEI

HDDTESAGKIGEEVNPVAAIKAEDVVCEIHAVGYEDRRKKGEYVPAGRKGHPEIEN

>XP_016444056.1

MEIDSTSKTDSKSKEQPGVFTRWWSQLKGFPRMLKDKSWNIAKDTKQIGKDDPRKIWHAAKVGLALTLVL

LFYYSWPLYHSFEQSAIVACLTVMVAFEYTAGATMSKCINIAFATALGGTLGIGAKYLAELCGKEGEPIV

LGFLVFILGALGTFTRFYPQMQRRYDYGCMLFVATFSLVTVSGDKYLDLDKQRISTIMVSVATVMIISLL

ICPVWAGEDLHNLVTTNLEKLASFLEEAEASGEGYKDNEKGFFEAFKSVLGSKATEESLANIAWWEPAHG

SFRFNHPWKQYLKIGGIARECAGHLQSLSGHLKSKSQAPIEFNRRTEKACKRMITESSKALKELAFSIKI

VTKLSTITTESHSYNARTAIADLKEALFTFKTFFLFEEADTIDVIGAMSVVSILIEVTKCVDKISEAVEE

LSIKARFNKEENNEKKKKKKKKNDSSSAKALEKPPPRPQLLIPVLEADNVNSGASVVIEIHDDTESAGKI

GEEVNPVAAIKAEDVVCEIHAVGYEDRRKKGEYVPAGRKGHPEIEN

>XP_016472383.1

MAYLGSLKQSFVETNKERILLPRKGYSELGVGGGASFTGNENFLERLRYRLTEFCNDVKKAAAKAVKMGR

SDPRKIIFSAKVGFALALVSILIFFKEPLPYIGKHSIWAILTVVVVFEFSIGATLSKGFNRALGTFSAGG

LALGIAELSLMAGKCQEVVIVISVFIAGFCATYLKLYPAMKQYEYGFRVFLLTYCIVLVSGTSDFVQTAV

SRLLLIGVGATVCLLINVCIYPIWAGEDLHKLVAKNFKGVATSLEGCVNDYLQCLEYERIPSKILLYQAS

DDPVYNGYRTAVESTNQEDSLLGFAVWEPPHGRYRMLNYPWGEYVKVSGALRHCAFTVMAMHGCILSEIQ

AASELRQVFRKQIQRVGTEGAKVIRQLGEKVEKMEKLFPGDPLEEVHEAAENLQLLIDQKSYLLVNAENW

ESSKRPKKFEDPERIQELKDNEPKPMLINSLSEATLHLRSAHTLKHMDTLNPNVSVNFSTSQWGSSEDVF

KQQTMWPSRLSVLGDVILNEREVRTFESASTLSLATFTSLLIEFVARLQNLVDAFQELSEKAKFKECN

>XP_016474426.1

MEIDSTNHEKAGIFTSWWSQLKGFPRKLKDKARNIAKNTKKIGKDDPRKIWHAAKVGLALTLVSFFYYNG

PLYHSFEQPVMWAVLTVVVVFEFTTGATISKSINRGTATALAGVFGVGAKYLAGLLGNEGPDPIVLGVLV

FIVGSVGTYTRFYPQIKRRYDYGTMIFVLTFSLVAVSSYRSEDILTVACERLATILIGVATVMTISMVIR

PVWAGDDLHKLVCTNLEKLASFLDGFGSEYFHIYEIEGNGGGTKSNEKGFLEAFISVIGSKATEESLANF

AWWEPPHGSFRISHPWKQYLKIGSLARECACHLVGLTGRLNSKSQVPTEFDRRTEEVCKRMITESSKVLK

ELALSIKTMTQPSSSFMEETHMRNAKSSIDDLKKTLGTSKTFFQNDESNVMDLVPAASVLSTLIDVTKCV

HEISKAVEELSVKADFKKKKDSPSSSSSSPPPPWSQLLHRGIVNPVMEDDVESGDFIVIEIGENIESAEK

VEIEEANPANNSLAAKKEESVVCEIHVVEEVKTEEKKRESVIIGVRESSAATTVEELKMAQKIMNFADIT

KLYDSTQFFEPVEIGVRGSTALVITTVMEDGIGGGESIPWRKGSSEIDNCK

>XP_016478809.1

MMEKRLNGVVERMKSTLPRLIWRKIWKVGKEDRRRVIHSLKMGLSLTLVSLLYLLEPLFKGIGENAIWAV

MTVVVVLEFTAGATLYKGLNRGLGTVFAGSLAFLIECIATESGHIFHAVFIGTSVFLVGAVATYMRFYPH

IKKNYDYGVVIFLLTFNLITVSSYRVDSVLKIAHERFYTIAIGCAICLLMSLLVFPIWSGEDLHISTVAK

FDGLAKSIEACINEYFSDKEEQEKAKENSMEAEDPIYNGYKAVLDSKSSDETLAVYASWEPRYLRRCSRL

PWQQYVKLGTLIRHFGYTLVALHGCLQTEIKTPRSVRVLFKDPCNQLAKEVTKVLKELGDSIRNRRQCCP

EMLSNHLNEALQGLINALKSQPRLFLGTNSNINILALTGAISSRQKSSKDFVVSLSSVNNIDTSNEGPAR

LGLGRDFLAGEAHGTTPALSKDTSKKDSEPQMKLEAGGRKSLRPTLSKIGITSLEFLEALPFAAFASLLV

ETVARLDLVIEEVVELGRIAHFKEYNPDDDHVAVITCDQNPRVEDEIVISRLQTQFPSSHAAD

>XP_016485525.1

MGVEMEVSGDNNTNWVPIMKKRMNILGKKMKNFPKITWKTIWKVGKDDPRRVIHSLKVGFSLTLVSLLYL

MEPLFKGFGSSAIWAVMTVVVVLEFTAGATLCKGLNRGLGTLLAGSLAFLIEYVATESGHIFRAIFIGAS

VFFIGAAATYMRFFPYIKKNYDYGVVIFLLTFNLITVSSYRVDNVLRIAHERFYTIAIGCGICLLMSLLI

LPNWSGEDLHNFTAAKFEGLAKSIEACVSEYFRDEEQQKARENSSDLEDPIYNGYKTVLDSKSFDETLAL

YASWEPRHSRHCYRFPWQQYVKLGNVLRHFGYTIVALHGCLQTEIQTPRSVRAMFKDPCIRLAGEVTKAL

KELGDSIRYRRQCSPEILSDHLHEALQDLDNAIKSQPKLFIGSKNNSNKLALATIGIQSGALNSRRVTLS

SVKTDTSALLEWKSKRGSTNENNERKSLRPTLSKIAITSLEFSDALPFAAFAALLVEIVARLDLVIEEVE

ELGRIAHFKEYNNSQDDDHNVVVDIEKHAMPLRNELPTHAGD

>XP_016490822.1

MEIDSTRKTDSKSKEQAGVFIRSWSQLKGFPRMLKDKAWNIAKNTKQIGKDDPRKIWHAAKVGLALTLVL

LFYYSWPLYHSFEQSAIMACLTVMVAFEYTAGATMSKCINIAFATALGGTLGIGAKYLAELCGKEGEPIV

LGFLVFILGAIGTFTRFYPHMQMRYDYGCMLFVATFSLVSVSGDKYLDLDKQRISTIMVSVGTVMIISLL

ICPVWAGEDLHNLVTTNLEKLASFLEGFGSEYFDVSEAEASGEGNKDNEKGYFEAFKSVLGSKATEESLA

NIAWWEPAHGSFRFNHPWKQYLKIGGIARECAGHLQSLSGHLKSKSQAATEFNRRTEKACKRMITESSKT

LKELAFSIKIVTKLSTITTESHTYNARTAIADLKETLFTFKTFFLFEEADTIDVIGAMSVVSILIDVTKC

IDKISEAVEELSIIARFNKEEKKEKKKKDDSSSAKALEKPPPRPRLLIPVLEDDNVNSTGASVMIEIHDC

TESAGKIAVEEVNPVAAIKADDVVCEIHAVEEVKIAEKR

>XP_016491147.1

MVTIKNFFKKASSDENKEKLLPNDYDERKGCCNCFSPLNDKFTSFFNNLQDFAKKAIEMGRNDPRKIIFS

LKMGFALSFVSLLIFWKKPNDIAQFAIWAILTVLVMFEFSIGATLIKGFNRGLGTFCAGMLAFIFAQLAL

WAGERERVVIVVSIFIVAFFGSYLKLYPTMAPYEYGYRVFILTYCILIVAGNRTREYNVAICTRLALIAL

GAGICLLINISIYPIWSGEDLHRLVVKNFMDLANSLEGCINGYLSCVEHDRDANDSEYNGYKSVIESTGR

EQTLLGFAIWEPPHGRYRMHKNLWRDFVKLSSALRHCAFMVMALHGCIQSEIQSPPEKRKVFRNELKRVG

ANAAKVLRELGTKLEKLEMLDSHGNILKEVHETAQQLQKKIDHKSYILVNSKSWEIGKPNINHEDSSSEN

GSENLPLSSRSRSLSETAIDISSLQANWPQLAKESSEFRNSSFRKQNQWPSRLSLADGDIADTCEMETYL

SASSLSLATFASLLIEFVARLQNVVDNFEELSQRAEFKEPDVIIPPSAIKS

>XP_016498527.1

MVKENEVSAASLEWRVNMPSGSSQILVPESRQTCRLLSLIMGLVSNIRKFFDKAWNLAVNEPKKVIHCLK

VGLALSIVSLFYYMRPLYDGVGGNAMWAVMTVVVVFEYTVGSTLYKCVNRAIGTCLAGSLGIGVHWVASQ

SGDRFEPVILQASVFLLAAAATFSRFIPTIKARFDYGAMIFILTFSLVSVSGYRVDKLVELAHERVSTIA

IGATICLFITMILCPVWAGTELHHLISTNLEKLADSLEGYAAENFRVDGSKNLDEKDSSKRLQGYKCVLN

SKAAEEAMANFARWEPAHGKFNFRHPWKQYLKIGASMRSCAYCIETLYGGINSNTETPEFLKKPLNDVCM

RLGTSSSKVLKELSSMLKTMTNSTKLDILVDEMNSSVEGLQNALKTLSSYQHIPPPDPKTEEAPNGTEES

ILKPTALSLMEIVQMATLTSLLIEIASRIEGIVKEVNELASQAKFRDESSKKSKQTQTKLNENDGNEHEV

TMTTLQKV

>XP_016510478.1

MAPVKNKENAGMLKVKGVVKKPVSKVYSFGSDMKKIAEDDPRKIVHSIKVGLTIALVSLFYYFEPFFHYE

GFGVSAMWAVLTVVVVFEFSVGATLGKSVNRGIATFLAGSLGVGAHKLVNLSGSDKLQPVVLGLSVFLIA

AIATFMRFVPKLKARYDYGILIFILTFSLISVSGYRDDEVLDMAITRVTTILIGGAAAVLFNVVICPVWA

GEDLHNLVATNIEKLGISLEGYGNQYFKKLDVKLEELDRTFLGEYKCIIHSKTNEVNLVNLAKWEPRHGK

FRYRHPWEQYLKIGDLVRDCAINIDALNTCLSSCIMTPEGKKIIQETCTKMSVECGCALKELALSMKTMI

IYPTTDSHILKAKAAAEKLRSIIRSGNLAEEAELRQLLPTTRVASLMLDFVSSSVEILDSVNHLAVLMKF

KILASKPKRLGSKNRIPSGNIGEAHIAINVE

>XP_024174659.1

MAQEKEMSRVTEWRIRMADGSSEALAPEAGLACRAWLALKGLIMGLVLKVCNFFRKAWDLGQNEPKKVIH

CLKVGMALTVVSLFYYMRPLYEGMGGNAMWAVMTVVVVFENTVGATICKSLNRICGTFVAGSLAIGIHWI

VSQSGKEFEPFINGISVFLLASAVTFSRFIPSVKKRFDYGAMIFILTFSLVAVSGYRVDKLLEMANNRVY

TIIIGTALCIMITMIIYPIWAGEELYMLITRNMDKLAYSLDGCVGEYFNESGSSSEGDKESAKKLLGYKC

VLTSKATEEAMANFARWEPAHGRFNFRHPWKQYLKIGASMRACAYCIDALDGCVNSENEVPELIKKHMSN

IALKVSSESSRVIKELAKTMKTMKKSSTIDYLVGEMNNAVLELQEDLKSLPTLFINPQPLQGADCPEKKN

TGAVALMDIMPLVTLVSLLIEIATRIEGLVNAVEELAEMGEYKSVADEKNQNQPVSKTNVPDEHKEGV

>XP_024172708.1

MFPKVHAGMDMEMNSNMDCGKSKGIIGEKSMRILVDKMKKYPVSIWRTMWKVGRDDPRRAIHAVKVGLAL

TLVSLLYLLQPLFTGIGQNAIWAVMTVVVVLEFTAGATLCKGLNRGLGTLLAGSLAFFIEYIATESGHIF

RAVFIGAAVFLIGAAATYIRFIPYIRKNYDYGVVIFLLTFNLITVSSYRVDNVMKIAHDRFFTIAIGCGV

CLLMSLLVFPNWSGEELHSSTVFKLEGLARSIEACVNEYFSEQDQIEANEDKSTKDPIYEGYKAVLDSKS

FDETMALHASWEPRHSRHCYRFPWQQYVKLGNVLRHFGYTIVALHGCLRTEIQTPRSVRGLFKDPCIRLA

GEVSKGLQELANSVRNRRHCSPEILSDHLHEALQDLNTAIKSQPRLFLGANSNQATNMLALAAAHATSQK

HAKYSSGGGGVSLSSVKTDSSALMEWKSKRESSERKFLRPQLSKIAITSLEFSEALPFAAFAALLVETVA

KLDNVIEEVEELGRIACFKEYEHGDENIVVTSDKPQIIDVTQNQLPSHGVD

>XP_024172498.1

MAAAKMGSFKRSFQEKKERLLSTNKRYSEVSFFPIEELEPYGSSSSSSSRWCCSFRSVSDKVAGWCRTVQ

GVSRRAIKMGQSDPRKIVFSAKMGLALMLISLLIFLKEPFKQLSRYSVWAILTVVVVFEFSIGATLSKGF

NRGLGTLSAGGLALGMAELSGLAGEWEEAVIVASIFIIGFIATYAKLYPTMKPYEYGFRVFLLTYCFIMV

SGYRTREFVHTAVSRFLLIALGAGVGLGVNILIYPIWAGEDLHKLVAKNFMGVAKSLEGCVSTYLNCIEY

ERIPSKILTYQASDDPLYSGYRSAVESTSQEDALMGFAIWEPPHGRYKMLRYPWKNYVKVSGALRHCAFT

VMALHGCVLSEIQAPAERRQVFSRELQRVGYEGAKVLCELGNKLKKMEKIGPIDILNEVHEAAEELQKKI

DQKSYLLVNSESWEIGNRPKELAEIQDLLDLDDEENKFHEYKSLSEAVLDLRSFPGSQSWDDPVPAKSAS

LNTSPPDSNPSNLPVPPGKMFMKQISWPAGMTFKAHAEPQVEESNTYQNASQLSLATFTSLLIEFVARLQ

NLVDSFEELGETALFKEPVDLPEPLESHGGFWTRLLDCLKL

>XP_024169760.1

MNGKKGSMEINIPCATTKVMKQHDIEANGGQKSGKKGGEGGESFSFRAWMRRVWDFIKEDSNRVKFAFKV

GLAVLLVSLLILFRAPYEVFGTNIIWSILTVAIMFEYTVGATFNRGFNRALGSLLAGILAIGVAQVALRS

GRVAEPVIIGISIFIVGAVTSFMKLWPSLAPYEYGFRVILFTYCLIVVSGYRISLGNPIRTAMDRLYSIA

IGGFVAVFVNVLVFPIWAGEQLHKELVNNFNSVADSLEECVEKYLEDDGAEHPEFSKTVMDEFPDEPAYR

KCRSTLNSSAKLESLAVSAKWEPPHGRFRHFFYPWSEYVKVAAVLRYCAYEVMALHGVLHSEIQAPYNLR

ITFQSEIKEATSQAAELVRSLGQDICNMKKSTRTSLLKKLHSSTEKLQRAIDIHSYLLTSSIDPPDSNNF

SKPLPKLPQTLSSTFSDLSNQLQAVQLDSTTSLEKNQSTQQQTAEPYHELMRKQSRRLHYSWPPRQVDAF

EDDGSAGSDFLPRMKALESTAALSLATFTSLLIEFVARLDHLVEAVDELSKLAKF

>XP_024163434.1

MGSTVISIPDGELSLPLPSIKKEKERPNKLSIIFSYLGELQKNRQKMRKLIHSIKVGIALVLVSLLYLLD

PLYEQVGENAMWAIMTVVVIFEFFAGATLSKGINRGLGTILGGGLGCSAATLAQEVTGMGEASTIIIGSA

VFVIGAAGTYTRLQPSIKKRYDYAAMIFILTFNLVVVSGLRAEEVLKLARERLSTIGMGFAVCIIISLLV

FPTWASDELHDSLSSKFQALAKAIEECLEDYFRLNSEKDNQPGQPSSSSGSCKSVLHSKSKDESLANFAK

WEPWHGKFGLHYPWNKYLQVGEQLRDLATIVLSLKACLQSPRQPSSSVRESIKEPSKAVGLSLALTLREL

GESVTKMRRSQQEAVIMPKLKSMRVELNSIISSSKFGPLENVDELAISSFVFLLMETVEKVEELVKEVEE

LGELADFGTK

>XP_024161316.1

MLKKLGQDDPRRIVHSLKVALALTLVSMLFYFEPLYEGLGLAAMWAILTVVVVFEFSVGATIGRGLNRMM

STVVAAALGIGAHRLATLTGNQGEPILIALFVFVEAGIVTFFRFIPQMKTRYDYGLLIFNVTFCLVSVSG

YRDKVVIQMGFERVCTITIGGCIAVAVCVFIRPVWIGVELHNRISTNMEKLANFLEEFGDEYFKVSENEQ

TREKSSFIEGYKSVLTSKGSENTMANLARWEPGHGNFRFRHPWEQYLKVGTLPRQCAFKIEALNNYLTSE

TQTPQEVKSIIQGPSVVISSECGKALKELATSVKKMTKSSSNEPHIAKSKDAAEELKAVIRSSLCKHPAD

SLHIIQEGAVASLLLEIITCIEKIADAAHKLASQAHFKDVKPTVKPDQENSVNDGPHHVITIEG

>XP_024161211.1

MMGSASNEKIGVLLASLTARFTGKVCEIADRTKKLGQDDPRRITHSLKVGFALTLVSLFYYYQPLYNSFG

ASAMWAVMTVIVVFEFSVGATLGKGLNRGLATLVAGALGIGAHHLASLSGHIGEPILLGIFVFLQAATST

FIRFFPKIKARYDYGLLIFILTFSLISVSGFRDDEVLELAHKRISTIFIGGSACVIVSILVFPVWAGEDL

HNLIAANIEKLGNFLEGFGGEYFKTLEDIAESKDDKAFLLGYKSVLNSKSSEESLANFARWEPGHGRFSF

RHPWTQYLKVGALIQQCSYRIEALHGRLITADIQASPEIRNRIEESCTKLSLESGKALKALASDFRTMTK

SPSADPHIANAKAAAKSLKSLLKSGLREDIDLLEVVPAATVASLLLDVVNCTEKIVESVYELGSLANFET

VEATVSPEKSHELGKICRDKKSPMMDCPHVVIKITESPPVLPVNIGKTSQQILEV

>XP_024161106.1

MASSNNDHDQVKGAGLFQRLRGKIVKFGLMLKKLGQDDPRRIVHSLKVALALTLISMLFYFEPLYDGFGI

AAMWAILTVVVVFEFTVGATIGRGLNRIMSTVVAAALGIGAHRLATLSGNQGEPILIGLFVFVEAGIVTF

FRFIPQMKARYDYGLLIFNLTFCLISVSGYRDEEVIRLGFERVSTIIIGSCTAVTVCVFIRPVWIGAELH

NQIATNMEKLANFLEEFGDEYFTVSENGQIRGKSSSLQGYKSVLASKGSEDTMANLARWEPGHGRFKFHH

PWKQYLKVGTLTRQCAFKIEALNNYLTSETQTPQEVKSIIQGPSVVISSECGKALKQLATSMRKMTKSSS

NDPHIANSKDAAEELKAVIRSSLCKLPAADSLHIIQEGAVASLLFEIIRCTEKIADAVHKLASQAHFNDV

KPTVTPNQENSVNDGPHHVIAIE

>XP_024159912.1

MASSNNDHDQVKSAGLFERLWGKIVKFGLMLKKLGQDDPRRIVHSLKVALAVTLVSMLFYFEPLYDGFGL

AAMWAILTVVIVFEFTVGATIGRGLNRMMATLVAAALGIGAHRLATLSGKQGEPILIALFVFVEAGIVTF

FRFIPQMKARYDFGLLVFNLTFCLISVSGYRDDEVIRMGFERTNTIIIGSCTAVAVCVFIRPVWIGVELH

NQIATNMEKLANFLEGFGDEYFSVSENSQNRDKSSSLQGYKSVLTSKSSEDTMANLARWEPGHGRFRFRH

PWKQYLKVGTLTRQCAFKIEALNNYLTSETQTPQDVKSIIQGPSVVISSECGKALKELATSMRKMTKSPS

KDPHIAKSKDAAEELKTVIRSSLCKHQDDSLDIIKDGAVALLLFEIITCTEKISEAAHKLASQAHFKDVK

PRVTPVQENSGNDGPHHVITIEG

>XP_024200591.1

MTSADFVDMENSSCGGGPFGRVVRWLKPVPERSWGKVLEAAREAKKLGEDDLRRIVHSFKVGLALTLVSL

FYYYRPLYDGFGDDAMWAVLTVVVVFEFSVGATLGKGINRMLATLLAGALAVGVHHISTLWGGEVGEPIL

IAFFVFVVAASVTFMRFFPALKARYDYGLLIFTLTFCLVSVSGYRETQVLDVAHKRLSTIAIGSCTSIMV

CICICPVWIGVDLHNLVAGNMENLGVFMEGFGFEYFDIKGNGLSLSNQQYKSVLSSKSLEETMVNLARWE

PGHGGFKFGHPWEKYLNVGSQTRQCAYKIEALNTYLNTGIQVPAEIRSEIQEPCINICTESGKALKELAA

ALKKMTRSSSADSHIACSKAAVESLKLLSLKRGIWKDADLLETIPSAAVASLLIEVVACVEKIAEAVHEL

ASVAHFKTEQPQLLQEPALSEEYHVAITIDRNSTEGLPKNGSSDCITTNV

>XP_024200590.1

MTPGPISPEDALKVNSNPSLISDNVVHDESNPKPHNVIHDIPNLEFNDVVHNEDAIRDEPYTYAIRDAIH

RALYIVKSEVTSADFVDMENSSCGGGPFGRVVRCLKAVPERSWGEVLEAASEAKKLGEDDPRRIVHSFKV

GLALTLVSLFYYCRPLYDGFGVDAMWAVLTVIVVFEFSVGATLGKGINMMLATLLAVALAVGVHHISTLW

GGEVGEPILIAFFVFVVAASVTFMRFFPALKARYDYGMLIFILTFCLVSVSGYRETQVLDMAHKRLSTIA

IGSCTPIMVCICICPVWIGVDLHNLVAGNMEKLGDFMEGFGFEYFDIKENGLSLSDNQYFLQQYKSVLSS

KSLEETMANLARWEPGHGGFKFGHPWQKYLNVGSQTRQCAYKIEALNTYLNTDIQVPTEIRSKIQEPCID

ICTESGKALKELAAALKKMIRSSSADSHIACSKAAVESLKLSLKRGIWKDANLLETIPSAAVASLLIEVV

ACVEKIAEAVHELASVAHFKTEQPQLLQEPVLSEEFNVAITIDRNSQI

>XP_024198112.1

MASQVAGVKNTNEDENAHKDLGFCGRVKNKVVEFPMKLKKLGRDDPRRIVHSLKVGLAVLLVSLLYYFDP

LYDGLGATAMWAVLTVVVVFEFSVGATLGRGLNRILATFLAGSLGFGVHHLANLSGEKGHPILIGLFVFI

LAASVTFFRFFPRIKARYDYGLLIFILTFCMISVSGYRDEEILEMAHKRASTILIGAFTAVSVCVFICPV

WAGDDLHNSVATNIEKLGSFLEGFGSECFKISCKGGESNKALLKGYCSVLNSKQSEESQVNFARWEPRHG

RFRFRHPWKHYLKIGSLTRQCAYRIDTLNGYINSEIQTPLNIHSTKVQELCMKISSESSKALKELALAMK

TMTRPTTATAHITKSRIAANSLKSFLKSPGLGGDIHLLDIIPAVTVASLLTDVVAYAEQIEKSIQELASL

SHVQFKSAEPKTEQEQTRLHKHGIVHPCAATDGPHHVITIVQPSLKEIENKG

>XP_024194679.1

MEKPLLVSFKEDEYERESSRYMCCFSFLGQKVVKFGEDVLDFALKAWEMGRSDPRKVIFAIKMGLALSIV

SLLIFWKKSFHDIDIGGTFIKGFNRGLGTLVAGILAFCFAKLSLLAGNLEEVIIVISVFIVGFFASYLKL

YPTMKPYEYGFRVFVLTYCILMVAGNRTREYKEAVTTRLVLIAVGVAVCLFVNICICPIWSGEDLHNLVV

KNFKEVATSLEGCVNGYLKFVEYERTPSKILTHQASDDPLYKGYRSVMESKNQEETLLGFAIWEPPHGRY

RMLNYPWKNYVKVSSALRHCAFMVMALHGCLLSEIQAPAEKRLVFRTELLRVGAEGAKVLRDLGKKIEKM

EKLGPGDILKDVHEAAENLQKSIDHRSYILVNSESWEIGRRPREQEDAENVSDSKDSDDMNLGFKSLSET

VLDLGSVSAAWTPSATQGASSECVFRNQPSWPLRLPFDGAGVSKEENCRTYESASALSLATFASLLIEFV

ARLQNVVDSLEELGEKAKFRELILNTPPTTKKSWIFCFI

>XP_024194192.1

MAAKIGSFRHSFAERSKERLLSRKGYSDFGLNSSECGDERVKCGWFGRMSDGLVNLCNSVQEVSVKLYQM

GRSDPRKAYFAAKMGLSLAIVSLLIFFKEPLKDASQYSIWAILTVVVVFEFSIGATLNKGFNRALGTISA

GGLALGIAELAVMAGSWQEVVIVVNIFIAGFLASYIKLYPPMKTYEYGFRVFLLTYCIVLVSGSTSSFFG

TAIYRLILIGVGAAVCLVINICILPIWAGEDLHKLVVKNFKGVATSLEGVVNGYLQCVEYERIPSKILTY

QASDEPLYNGYRATIQSSTQEETLLDFALWEPPHGPYRSFHYPWRNYVRVGGSLRHCAFMVMAMHGCILS

EIQAPPEKRQVFADELLRVGTEGAKVLRELGSKVERMEKLSPKDILFEVHEAAEDLQNKIDQKSYLLVNY

EGWGPEILGRKFEDPEQDTRNNVIESLSESWDAQNIGGGGGDPSMSQWISTDSVLKKTLTAWPSRLTSFT

GNLMQPQPELEESKVYESASSLSLATFTSLLIEFVARLQNLVDEFVELSEKANFKDPMDVSDTKEVGGFW

TRIYRRWWSKNDELA

>XP_024190282.1

MRVKCGWFGKMSDGLVEFYNSVQEVSVKLYQMGRSDPRKAYFAAKMGLSLAIVSLLIFFKEPLKDASQYS

ILAILTVVVVFEFSIGATLNKGFNRALGTISAGGLALGIAELAAMAGTGRRCDCSQYWISSNALQMDLPL

AWLCFFGDLMQMNIVLGVIICLVFLHQNGAAIQSPHTAL

>XP_024188011.1

MAQEKEMSRVTEWRIRMADGSSEALAPEAGLACRAWLALKGLIMGLVLKVCNFFRKAWDLGRNEPKKVIH

CLKVGMALTVVSLFYYMRPLYEGMGGNAMWAVITVVVVFENTVGATICESLNRICGTFMAGSLAIGIHWI

VSQSGKEFEPFINGISVFLLASAVTFSRFIPSVKKRFDYGAMIFVLTFSLVTVSGYGRVDKLLEMTNDRV

YTIIIGTSLCIMITMIICPIWAGEGLYMLITRTWTNLHIRCVGEYFNDSRSSSDGDKESDKKLLGYKCVL

TSKATEETMKDITNAYLQLATPSRQQVPELIKKHMSNIALKVSSESSRVIKELARTMKTMKKSSTIDYLV

GEMNNAVLELQEDLKSLPNLFINPQPLQGADCPEKRNPGVVALMDIMPLVTLVSLLTEIATRIEGMVNAV

EELAEMGEYKSIADAKNQNQPVSKTNVPADEHKEKV

>XP_024186382.1

MEIINDQLAGGEHRKARVFERAWSWVQALPGKLKNKVTKVAKNTIKLGKDDPRRVIHSLKVAMALTLVSL

LYYWRALYDGLGVAGMWAVLTVVVVFEFTVGATLSKGLNRSFATLLAGALGIGANHLAGLFGEQGEPIIL

GVLVFLLAAASTFSRFFPRIKARYDYGVLIFILTFSMVTVSGYRVEGLLEMADQRLVTVLIGGGTCIIIA

IFLCPVWAGEDLHNLIASNTDKLANYLEGIGGGEYFQLPKDGKSTKVIKNDGYKSVLNSKTTEDSWANFA

RWEPGHGRFKFCHPWKQYLKIGALVRQCAYQIDTLNGHINSDVHVPQEFLQIVQASCITMSTESGKALKA

LGTAIKTMKDPKSACQHLETSKNAVEDLHIALKAASLENADVLAIIPAATVASILVEIVKCVEKISTSVH

ELSQQAHFKVAEPTVSPENKPQLLHRGSINPLLLDDGDDSAHVVITVDEMGSQKFSSGAKSPKTK

>XP_024176279.1

MVNAKDVPENLEWSINVTDGTKQVVAVPELRLVNKVWLGSKSVVGGLILTVWRFLVKAWNLGVAEPKKVI

HAVKVGLALTIVSLCYYIRSLYEGVGGNAMWAVMTVVVVFESSVGATLYKSINRVTATFLAGSLGLGVHW

ISYKAGEKFEPIIIGISVFFLASAATFSRFIPSVKSRFDYGALIFILTFSLVSVSGYRVDGISELAFQRL

STIFIGTSFCILISILFYPTWAGAELHRLIYRNLEKLADSLDGYVIEYFDYEAVAEDSNHTKAMKGYKYV

LDSKATEDNLAKFARWEPAHGCFNFQHPWKQYLKIGASIRSSAYCIEALSGCMDSEIQAPGIGYLKKHVS

NACKTMNKFSSDILRELAITLKTMTKSSQMGSLVREMNIAVQQLQISLRSIPNCLIAPTSEDTTDEPSRK

TCITPLVEVIPLTTFVSLLIENAARINGIVDAVNELAGMVDMKHGNSNAKTTQ
